# Long-Range Temporal Correlations, Multifractality, and the Causal Relation between Neural Inputs and Movements

**DOI:** 10.3389/fneur.2013.00158

**Published:** 2013-10-09

**Authors:** Jing Hu, Yi Zheng, Jianbo Gao

**Affiliations:** ^1^Institute of Complexity Science and Big Data Technology, Guangxi University, Nanning, China; ^2^PMB Intelligence LLC, Sunnyvale, CA, USA

**Keywords:** brain-machine interface, Fano factor, adaptive fluctuation analysis, wavelet, neuronal firings

## Abstract

Understanding the causal relation between neural inputs and movements is very important for the success of brain-machine interfaces (BMIs). In this study, we analyze 104 neurons’ firings using statistical, information theoretic, and fractal analysis. The latter include Fano factor analysis, multifractal adaptive fractal analysis (MF-AFA), and wavelet multifractal analysis. We find neuronal firings are highly non-stationary, and Fano factor analysis always indicates long-range correlations in neuronal firings, irrespective of whether those firings are correlated with movement trajectory or not, and thus does not reveal any actual correlations between neural inputs and movements. On the other hand, MF-AFA and wavelet multifractal analysis clearly indicate that when neuronal firings are not well correlated with movement trajectory, they do not have or only have weak temporal correlations. When neuronal firings are well correlated with movements, they are characterized by very strong temporal correlations, up to a time scale comparable to the average time between two successive reaching tasks. This suggests that neurons well correlated with hand trajectory experienced a “re-setting” effect at the start of each reaching task, in the sense that within the movement correlated neurons the spike trains’ long-range dependences persisted about the length of time the monkey used to switch between task executions. A new task execution re-sets their activity, making them only weakly correlated with their prior activities on longer time scales. We further discuss the significance of the coalition of those important neurons in executing cortical control of prostheses.

## 1 Introduction

Brain-machine interface (BMI) is aimed to provide a method for people with damaged sensory and motor functions to use their brain to control artificial devices and restore their lost ability via the devices. The feasibility of using adaptive input-output models to map the fundamental timing relations between neural inputs and hand movement trajectory has been extensively demonstrated (1–20). To achieve the mapping, model parameters are chosen in such a way that the difference between model output and hand movements is minimized using a statistical criterion such as mean-square error (21–24). The adaptive models proposed usually contain a very large number of parameters and require very extensive training (25–27). Moreover, they assume that neuronal firings in the cortex are stationary, while in reality this rarely can be true. This limits the optimal correlation between model output and hand trajectory to be around 70–80%. To help gain fundamental understanding of the causal relation between neural inputs and hand movements, in this study, we examine long-range temporal correlations (or long-range dependence, LRD in short) and multifractality in a large group of neuronal firings.

Events in extracellular neuronal recording generate two types of time series: (1) the time interval between successive firings, called the inter-spike interval (ISI) data; (2) a counting process, representing the number of firings in a chosen time window. In the last two decades, considerable efforts have been made to characterize fractal, non-Poisson behavior of neuronal dynamics (28–38). While most early works along this line employ Fano factor analysis, recently, other techniques, including detrended fluctuation analysis (39) and multiplicative cascade multifractal (40) have also been used. In this work, we employ three different types of fractal and multifractal analysis methods to explore how temporal LRD may be associated with the causal relations between neural inputs and movement trajectory.

## 2 Materials and Methods

### 2.1 Experimental procedures

Neuronal firing data for 104 cells, collected synchronously at Duke University when an owl monkey performed a three-dimensional (3-D) reaching task involving a right-handed reach to food and subsequent placing the food to mouth (11) were analyzed here. The total observation time was about 36 min. While the details of the behavioral paradigm and surgical procedure for chronic microwire recordings can be found in the literature (11), it is important to mention the components of the paradigm that are important for this work. Microwire electrodes were implanted in the cortical regions with known motor associations (41). Table 1 shows the assignment of electrode arrays in the four cortical regions. The monkey’s hand position, which was considered as the desired signal by adaptive models, was also recorded (with a time shared clock) and digitized with 200-Hz sampling rate. On average, the time interval between two successive reaching tasks is about 8 s. From the neuronal firing data, spike detection was performed. Note that some neurons fired more than 10^4^ times during about 36 min, while a few neurons only fired a few tens of times during this entire time period. This indicates the tremendous differences among the neurons. This point will be further elaborated through statistical analysis and information theoretical analysis below.

**Table 1:** **Assignment of electrode arrays in the four cortical regions**.

Area 1 33 Cells	Area 2 31 Cells	Area 3 27 Cells	Area 4 23 Cells
Left posterior parietal (PP)	Left primary motor (MI)	Left dorsal premotor (PMD)	Right primary motor and dorsal premotor (MI/PMD)

Areas 1–3 were contralateral, and Area 4 ipsilateral, to the moved limb.

### 2.2 Statistical and information theoretical analysis

If a neuron fires according to a Poisson process, then its ISI follows an exponential distribution described by

(1) $$f_{X}\left(x\right)=\lambda e^{-\lambda x},\;x\geq 0$$

where λ > 0 is a parameter. To assess how different the distribution of ISI is from an exponential distribution, we shall also examine gamma distribution, log-normal distribution, and power-law distribution.

The gamma distribution is specified by

(2) $$f\left(x\right)=\frac{1}{\Gamma \left(t\right)}\lambda^{t}x^{t-1}e^{-\lambda x},\;x\geq 0$$

where parameters λ, *t* > 0, and Γ(*t*) is the gamma function:

$$\Gamma \left(t\right)=\int_{0}^{\infty }\;y^{t-1}e^{-y}dy.$$

When *t* is an integer (say *k*), the distribution is called the Erlang distribution, which governs the summation of *k* independent exponentially distributed random variables.

Log-normal distribution is given by

(3) $$f\left(y\right)=\frac{1}{\sigma y\sqrt{2}\pi }e^{-\frac{{\left(\text{ln}y-\mu \right)}^{2}}{2\sigma^{2}}}$$

It is the distribution for the random variable *Y* = *e^X^*, where *X* has a normal (or Gaussian) distribution. To assess the goodness-of-fit of log-normal distribution to certain ISI data, one can first take logarithm of the ISI data, then check if the distribution is similar to Gaussian.

Power-law distribution can be written as

(4) $$P\left[X\geq x\right]={\left(\frac{b}{x}\right)}^{\alpha },\;x\geq b>0,\;\alpha >0$$

where α and *b* are called the shape and the location parameters, respectively. When plotted in double-logarithmic scale, such a distribution produces a straight line. To better understand the causal relation between neural inputs and movement trajectories, it is useful to analyze the correlations between them. One simple way is the cross-correlation analysis. More general correlations, including non-linear correlations, can be characterized by mutual information. To compute the dependence of cross-correlation and mutual information with time, one can partition the data into many small segments, then calculate the correlations between the corresponding segments, and finally plot the correlation against the time index associated with each segment. Let the segment of firing data of a neuron be denoted by *w*(*t*), and hand trajectory (either *x*, *y*, or *z* component) be denoted by *u*(*t*). The cross-correlation, denoted by *C*(*w*,*u*), can be calculated by the simple equation

$$C\left(w,u\right)=max_{L}\left\langle w\left(t\right)u\left(t-L\right)\right\rangle ,$$

where ⟨⟩ denotes average within the segment, and *L* is a small time chosen in such a way that ⟨*w*(*t*)*u*(*t* − *L*)⟩is maximized. When *C*(*w*,*u*) is normalized by the standard deviations of *w*(*t*) and *u*(*t*), one obtains the correlation coefficient. Before taking the average within each segment, one could remove the mean values of *w*(*t*) and *u*(*t*) first.

The mutual information of *w*(*t*) and *u*(*t*), written as *I*(*w*,*u*), is the amount of information gained about *u* when *w* is learned, and vice versa. Denote the probability distribution for *w*(*t*) by *P*(*W* = *w_i_*), *i* = 1, …, *N_w_*, that for *u*(*t*) by *P*(*U* = *u_i_*), *i* = 1, …, *N_u_*, and the joint distribution for *w*(*t*) and *u*(*t*) by *P*(*W* = *w_i_*, *U* = *u_j_*). Then

(5) $$\begin{array}{ccc} I\left(W,U\right) & =H\left(U\right)-H\left(W|U\right)=H\left(W\right)-H\left(U|W\right) & \\ & =H\left(W\right)+H\left(U\right)-H\left(W,U\right)\\ & =\sum_{i=1}^{N_{w}}\sum_{j=1}^{N_{u}}P(W=w_{i},\;U=u_{j})\text{ln}\\ & \;\frac{P\left(W=w_{i},U=u_{j}\right)}{P\left(W=w_{i}\right)P\left(U=u_{j}\right)} \end{array}$$

*I*(*w*,*u*) = 0 if and only if *W* and *U* are independent.

### 2.3 LRD and multifractal analysis

Multifractal theory provides an elegant statistical characterization of many complex dynamical variations in nature and engineering (42). There are two major types of multifractal analysis, structure function based and singular measure based (42, 43). For a relation between them, we refer to Hu et al. (44).

Within the framework of structure function based multifractal analysis, there are three techniques (42): one is the standard approach, including the Fano factor analysis; another is detrending based; the third is wavelet based. While they are equivalent when analyzing ideal fractal processes, detrending and wavelet based formulations are more reliable when analyzing real world data (43). To facilitate interpretation of our analysis below, we describe them in order here.

#### 2.3.1 Structure function based multifractal analysis

Let *X* = {*X_t_*:*t* = 0, 1, 2, …} be a covariance stationary stochastic process with mean μ, variance σ^2^, and autocorrelation function *r*(*k*), *k* ≥ 0. The process is said to have long-range correlation or LRD (42) if *r*(*k*) is of the form

(6) $$r\left(\pi \right)∼k^{2H-2},\;\;as\;\;k\to \infty ,$$

where 0 < *H* < 1 is the Hurst parameter: depending on whether *H* is smaller than, equal to, or larger than 1/2, the process is said to have anti-persistent, short-range, or persistent long-range correlations (42, 43). Note that when 1/2 < *H* < 1, Σ*_k_* *r*(*k*) = ∞. This justifies the term “long-range correlation” or LRD. We now consider estimation of *H*. A convenient framework is based on the random walk process *y*, defined as,

(7) $$y_{k}=\sum_{i=1}^{k}\;\left(X_{i}-\bar{X}\right),$$

where $\bar{X}$ is the mean of *X*. We then examine whether the following scaling laws hold or not,

(8) $$F^{\left(q\right)}\left(m\right)={\left\langle |y\left(i+m\right)-y\left(i\right){|}^{q}\right\rangle }^{1∕q}∼m^{H\left(q\right)},$$

where *q* is real and the average is taken over all possible pairs of (*y*(*i* + *m*), *y*(*i*)). Note that *q* > 0 emphasizes large absolute value, while *q* < 0 emphasizes small absolute value (to better understand this statement, it is helpful to think about concrete cases such as *q* = 10 and −10). When *H*(*q*) is a constant, the process is called a monofractal; otherwise, it is called a multifractal. The case of *q* = 2 is of special interest, since *H*(2) = *H*. In this case, equation (8) is often called fluctuation analysis (FA). It is equivalent to many other methods, including Fano factor analysis. In the context of ISI analysis, this can be explained as follows. Fano factor is defined as

(9) $$F\left(T\right)=\frac{Var\left[N_{i}\left(T\right)\right]}{Mean\left[N_{i}\left(T\right)\right]}$$

where *N_i_*(*T*) is the number of spikes in the *i*th window of duration *T*. For a Poisson process, *F*(*T*) is 1, independent of *T*. For a fractal process, *Var*[*N_i_*(*T*)] ∝ *T*^2^*^H^*, while *Mean*[*N_i_*(*T*)] ∝ *T*. Therefore,

(10) $$F\left(T\right)∼T^{2H-1}$$

In other words, Fano factor can be viewed as examining the relation between [⟨|*y*(*i* + *m*) − *y*(*i*)|^2^⟩/*m*] and *m* instead of the relation between [⟨|*y*(*i* + *m*) − *y*(*i*)|^2^⟩] and *m*.

#### 2.3.2 Multifractal adaptive fluctuation analysis

When a time series is non-stationary or containing a trend, the standard structure function based approach does not work well. Our experiences with fMRI analysis (45, 46) and other applications (47) suggest that detrended fluctuation analysis (DFA) and wavelet multi-resolution analysis are more robust. Here we apply another powerful method, adaptive fluctuation analysis (AFA), which is similar to DFA but provides additional advantages over DFA (48, 49). For example, AFA can deal with arbitrary, strong non-linear trends while DFA cannot (44, 50), AFA has better resolution of fractal scaling behavior for short time series (51), AFA has a direct interpretation in terms of spectral energy while DFA does not (50), and there is a simple proof of why AFA yields the correct *H* while such a proof is not available for DFA (see equations (6) and (7) in Ref. (50)).

AFA works as follows (50). We first construct a random walk process from the original data using equation (7). Next, for a window size *w*, we determine, for the random walk process *u*(*i*) (or the original process if it is already a random walk process), a global trend *v*(*i*), *i* = 1, 2, …, *N* (44, 52, 53). Here *N* is the length of the random walk process. The residual, *u*(*i*) − *v*(*i*), characterizes fluctuations around the global trend, and its variance yields the Hurst parameter *H*,

(11) $$F^{\left(2\right)}\left(w\right)=\left[\frac{1}{N}\sum_{i=1}^{N}\;{\left(u\left(i\right)-v\left(i\right)\right)}^{2}\right]∼w^{2H}$$

AFA can be easily extended to MF-AFA, by extending equation (11) to a multifractal formulation,

(12) $$F^{\left(q\right)}\left(w\right)=\left[\frac{1}{N}\sum_{i=1}^{N}\;|u\left(i\right)-v\left(i\right){|}^{q}\right]∼w^{qH\left(q\right)}$$

where *q* is a real number: depending on whether *q* is positive or negative, large or small values of deviations are emphasized, respectively. In many applications, the case of *q* = 2 may be most concerned, since *H*(2) = *H*. For notational convenience, *F*^(2)^(*w*) may be simply denoted as *F*(*w*).

Equation (11) can also be extended to high-dimensional case, such as an image or a high-dimensional trajectory. In the case of 2-D, this can be achieved by first applying the algorithm to the *x*-component of the data, then applying it to the *y*-component. At least for the polynomial order 1, whether *x*-component first or *y*-component first will not make any difference, so far as functions such as *d/dx d/dy f*(*x*,*y*) = *d/dy d/dx f*(*x*,*y*).

#### 2.3.3 Wavelet based multifractal analysis

The essence of wavelet based multifractal analysis is similar to that of adaptive multifractal analysis. Technically, it is based on the coefficients of a discrete wavelet decomposition. It involves a scaling function ϕ_0_ and a mother wavelet ψ_0_. The scaling function satisfies

$$\int_{-\infty }^{\infty }\;\phi_{0}\left(n\right)dn=1.$$

The wavelet ψ_0_ must have zero average and decay quickly at both ends (54). The scaled and shifted versions of ϕ_0_ and ψ_0_ are given by

$$\begin{array}{llll} \phi_{j,k}\left(n\right) & =2^{-j∕2}\phi_{0}\left(2^{-j}n-k\right),\; & & \;\\ \psi_{j,k}\left(n\right) & =2^{-j∕2}\psi_{0}\left(2^{-j}n-k\right),j,k\in Z,\; & & \; \end{array}$$

where *j* and *k* are the scaling (dilation) and the shifting (translation) index, respectively. Different value of *j* corresponds to analyzing a different resolution level of the signal. One popular technique used to perform the discrete wavelet transform (DWT) is the multiresolution analysis (MRA). The procedure of performing MRA consists of the following steps (54):

(1) At the *j*-th resolution, compare ϕ*_j_*_,_*_k_*(*n*) and ψ*_j_*_,_*_k_*(*n*) to the section at the start of the input signal *x*(*n*), this amounts to taking *k* = 0. Calculate the approximation coefficient *a_x_*(*j*,*k*) and the detailed coefficient *d_x_*(*j*,*k*) using the following equations
  $$\begin{array}{llll} a_{x}\left(j,k\right) & =\sum_{n}\;x\left(n\right)\phi_{j,k}\left(n\right)=\sum_{n}\;x\left(n\right)2^{-j∕2}\phi_{0}\left(2^{-j}n-k\right)\; & & \;\\ d_{x}\left(j,k\right) & =\sum_{n}\;x\left(n\right)\psi_{j,k}\left(n\right)=\sum_{n}\;x\left(n\right)2^{-j∕2}\psi_{0}\left(2^{-j}n-k\right)\; & & \; \end{array}$$
(2) Shift ϕ*_j_*_,_*_k_*(*n*) and ψ*_j_*_,_*_k_*(*n*) to the right, this corresponds to taking *k* = 1, 2, …. For each *k* value, repeat step (1) until the whole signal is covered.
(3) The signal approximation *SA_j_* and the signal detail *SD_j_* at the *j*-th resolution level are computed as
  $$\begin{array}{llll} & SA_{j}=\sum_{k}\;a_{x}\left(j,k\right)\phi_{j,k}\left(n\right)\; & & \;\\ & SD_{j}=\sum_{k}\;d_{x}\left(j,k\right)\psi_{j,k}\left(n\right)\; & & \; \end{array}$$
(4) Repeat steps (1) through (3) for the (*j* + 1)-th resolution level but use the signal approximation *SA_j_* obtained in step (2) as the input signal.

For examples detailing these steps, we refer to Hu et al. (47) and Gao et al. (42).

Let the maximum scale resolution level chosen for analysis be *J*. The signal can be reconstructed using the following equation (54):

(13) $$\begin{array}{llll} x\left(n\right)=SA_{J}+\sum_{j=1}^{J}\;SD_{j} & =\sum_{k}\;a_{x}\left(J,k\right)\phi_{J,k}\left(n\right)\; & & \;\\ & +\sum_{j=1}^{J}\;\sum_{k}\;d_{x}\left(j,k\right)\psi_{j,k}\left(n\right). \end{array}$$

The first term represents the approximation at level *J*, and the second term represents the details at resolution level *J* and lower. MRA builds a pyramidal structure that requires an iterative application of the scaling and the wavelet functions, respectively. Let

$$\Gamma \left(j\right)={\left[\frac{1}{n_{j}}\sum_{k=1}^{n_{j}}\;|d_{x}\left(j,k\right){|}^{2}\right]}^{1∕2},$$

where *n_j_* is the number of coefficients at level *j*. For fractal signals such as the spike counting process, one has

(14) $$\log_{2}\Gamma \left(j\right)=\left(H-1∕2\right)j+c_{0},$$

where *c*_0_ is some constant.

Formulation based on equation (14) can be extended to wavelet based multifractal analysis, by considering

(15) $$\gamma_{j}\left(q\right)={\left[\frac{1}{n_{j}}\sum_{k=1}^{n_{j}}\;|d_{x}\left(j,k\right){|}^{q}\right]}^{1∕q},$$

and examining whether the following scaling relations hold or not:

(16) $$\gamma_{j}\left(q\right)∼2^{j\left[H\left(q\right)-1∕2\right]}.$$

If yes, then we have

(17) $$\log_{2}\gamma_{j}\left(q\right)=\left[H\left(q\right)-1∕2\right]j+c_{q}$$

where each *c_q_* is some constant.

## 3 Results

### 3.1 Varying degree of correlation between neuronal firings and hand trajectory

**Figure 1:**
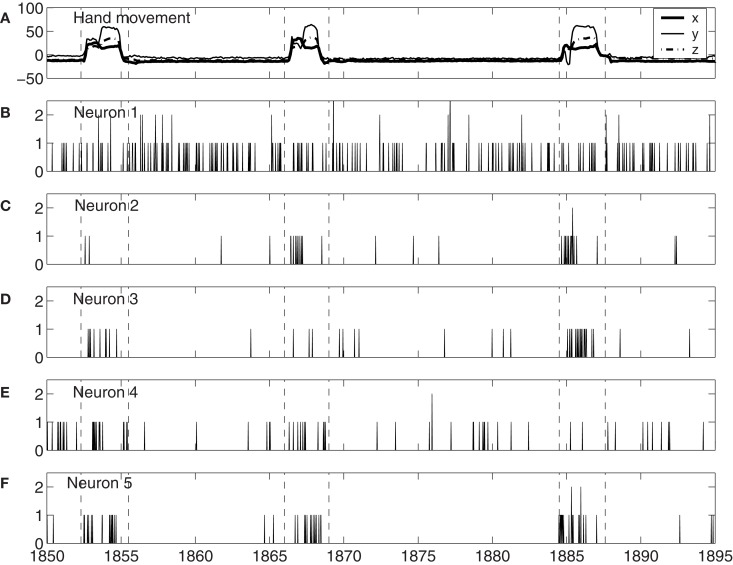
**(A)** *X*, *Y*, *Z* components of the monkey’s hand movements. Dashed lines indicate time intervals when the monkey stretched its hand to grab food and subsequently place the food to its mouth. **(B–F)** Neuronal firings of five neurons associated with the hand movements plotted in **(A)**.

To understand how neuronal firings control hand trajectory, it is instructive to visually examine the correlation between neural firings and hand trajectory. For this purpose, three consecutive hand movements are shown in Figure 1A, while neuronal firings of five neurons associated with those three hand movements are shown in Figures 1B–F. A number of interesting features can be observed from Figures 1B–F: (i) the firing rate varies considerably among the neurons. For example, neuron 1, plotted in Figure 1B, fired a lot more than most other neurons. (ii) The firing of neuron 1 of Figure 1B does not have much correlation with the hand trajectory. In fact, more than half of the neurons behaved like this. (iii) While neurons 2–4, plotted in Figures 1C–E, appear to have strong correlations with the hand movement trajectory, the degree of correlation varies with time considerably. For example, neuron 2, 3, and 4 did not fire much during the monkey’s “first,” “second,” and “third” period of hand movement, respectively. It should be mentioned that albeit neuron 5, shown in Figure 1F, fired a lot during all these three periods, it also had “quiet” periods even though the monkey was actively grabbing food to mouth. These observations suggest (i) different neurons have different degree of importance in determining the causal relation between neural inputs and hand movements, and (ii) even for the same neuron, this degree of importance varies with time considerably.

### 3.2 Heterogeneity of neuronal firings revealed by distributional analysis

**Figure 2:**
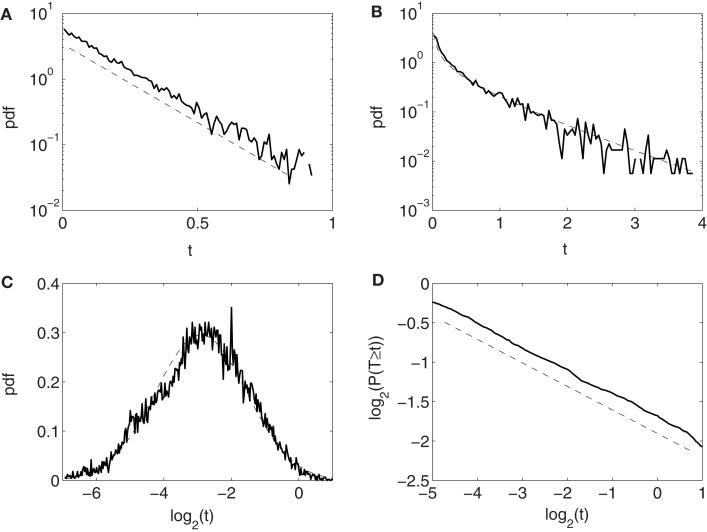
**Four types of neuron ISI distributions**. **(A)** Exponential, **(B)** gamma, **(C)** log-normal, and **(D)** power-law. Plotted in **(A–C)** and **(D)** are probability density functions (pdfs) and complementary cumulative distribution function (CCDF), respectively.

Conventionally, neuronal ISI data are modeled by exponential and gamma distributions. Besides these two distributions, many other distributions have been observed from the monkey’s neuronal firing data, such as log-normal and power-law distributions. Four examples are shown in Figures 2A–D, for exponential, gamma, log-normal, and power-law distributions, respectively, for four different neurons. Systematic distributional analysis of the 104 neuronal firings examined here reveals that there are 13, 21, 24, and 22 neuronal firings follow exponential, gamma, log-normal, and power-law distributions, respectively, while 24 firings could not be classified, due to lack of data. See Table 2. These distributional analyses clearly indicate that the ISI data of different neurons may follow very different distributions, and therefore, the neurons, in terms of their firing patterns, can be considered very heterogeneous. It is well-known that associated with a distribution, there is a specific stochastic process (55, 56). Existence of multiple distributions therefore implies existence of different stochastic processes underlying neuronal firings in the cortex.

**Table 2:** **Number of neuronal firings following exponential, gamma, log-normal, and power-law distributions**.

Distribution	Exponential	Gamma	Log- normal	Power- law	<100 Spikes
Number of neurons	13	21	24	22	24

When fewer than 100 spikes had occurred, we did not attempt to classify the distribution.

### 3.3 Non-stationary neuronal firings revealed by correlation analysis

**Figure 3:**
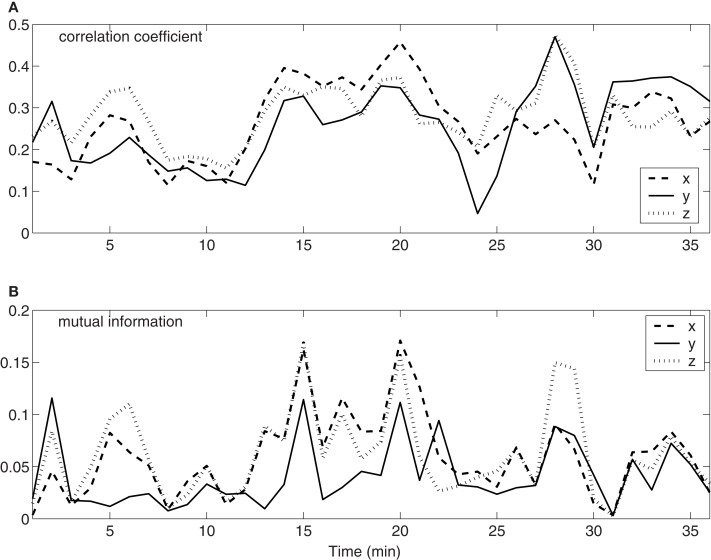
**Time-varying correlations between spike counting data and hand movement data**. **(A)** Correlation coefficient; **(B)** mutual information.

To quantify the time-varying correlations between the neuronal firings and movement trajectory, we computed cross-correlation coefficient and mutual information. The results are shown in Figures 3A,B, respectively. Evidently, both types of correlations vary with time considerably. Since hand trajectory is stationary, this indicates that neuronal firing patterns are highly non-stationary. The non-stationarity is one of the fundamental reasons that the accuracy of currently used adaptive models cannot be further improved.

### 3.4 LRD in neuronal firings revealed by Fano factor analysis

**Figure 4:**
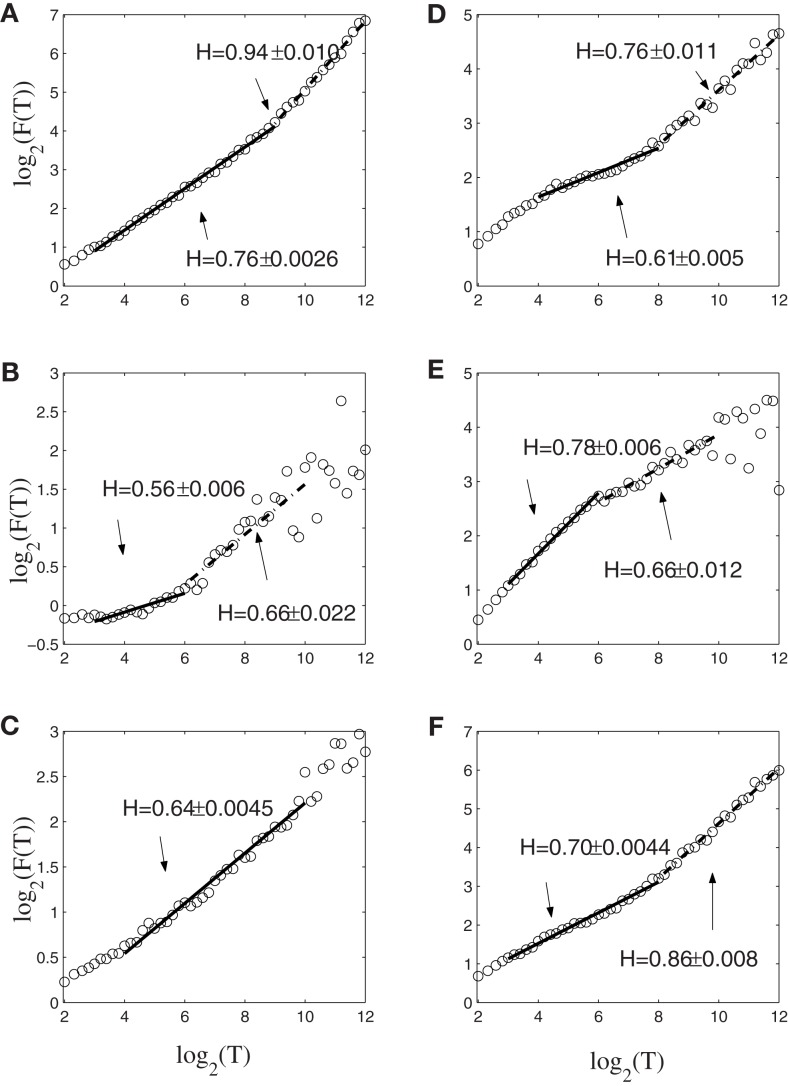
**Fano factor analysis of the spike count data of 6 neurons, where *F*(*T*) denotes the Fano factor**. The slopes in the figure amount to 2*H* − 1. Note that visually, neurons **(A–C)** are not well correlated with hand movements, while neurons **(D–F)** are highly correlated with hand movements.

To gain insights into the distinguished features that define the set of neurons that have strong correlations with hand trajectory (i.e., neurons that are similar to those shown in Figures 1C–F), we now carry out Fano factor analysis on the counting process of spikes. For this purpose, we have classified neurons into two groups, one group is not correlated with hand movement, just as the one shown in Figure 1B, while the other group is well correlated with hand movement, as those shown in Figures 1C–F. We then have obtained the counting processes by choosing the size of the small time window to be Δ*t* = 0.1 s. Figure 4 shows the results for six neurons, where *F*(*T*) denotes Fano factor. Note that neurons (a–c) are not well correlated with hand movement, while neurons (d–f) are. Recall that for a Poisson process, *F*(*T*) = 1, while for a fractal process, *F*(*T*) ∝ *T*^2^*^H^*^ − 1^. Since in no cases *F*(*T*) = 1, we have to conclude that Poisson processes are not relevant here. While Figure 4 suggests fractal neuronal firings, we have to note that the power-law scaling of *F*(*T*) ∝ *T*^2^*^H^*^ − 1^, where 2*H* − 1 is the slope of the plots in the Figure, are not well defined for most of the neurons. Nevertheless, *H* is generally greater than 1/2, especially when time scale is large. Recall that *H* > 1/2 indicates LRD. In the context of neuronal firings, this means that very active firing will be more likely followed by another active firing, while quiet firing will be more likely followed by another quiet firing. In other words, a small ISI will be more likely followed by another small ISI, while a large ISI will be more likely followed by another large ISI [for a similar interpretation in the context of switching times in multistable visual perception, we refer to (57, 58)]. Therefore, the firings of these neurons have LRD. However, Fano factor analysis does not clearly indicate the correlations between neuronal firings and the hand movement.

### 3.5 LRD in neuronal firings revealed by AFA

**Figure 5:**
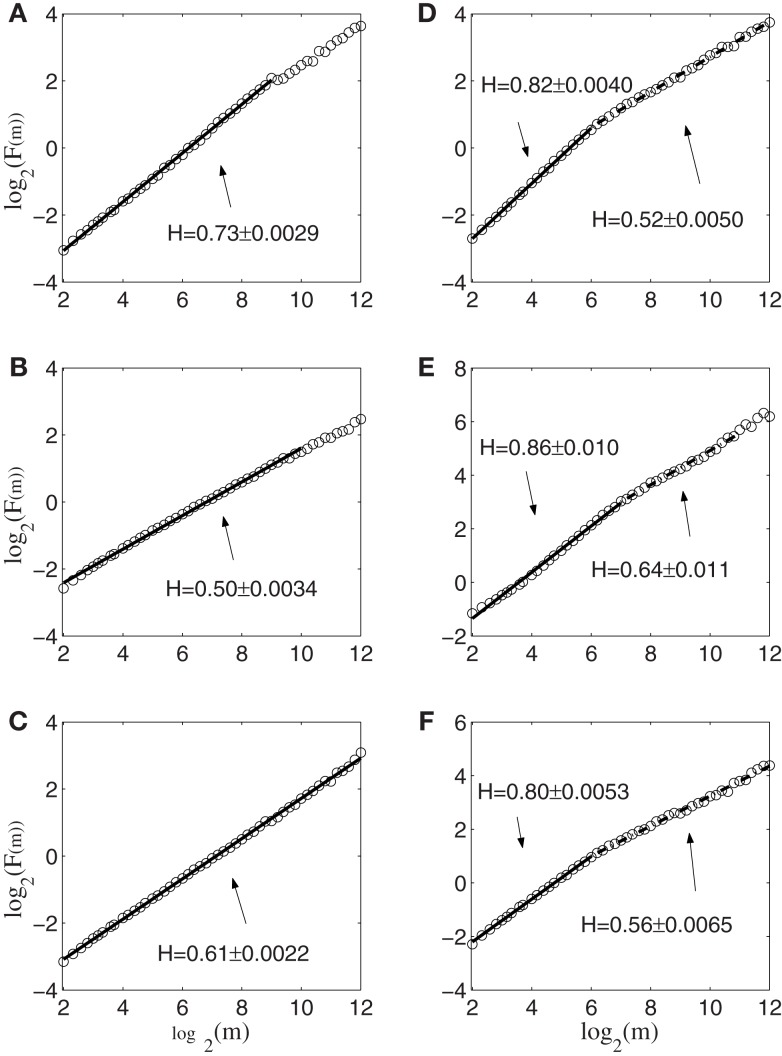
**AFA of the same six neurons**. Note that the slopes in the figure amounts to 2*H*. Also note that neurons **(A–C)** only have one scaling range, while neurons **(D–F)** have two scaling ranges.

Next, we apply AFA to the spike counting processes of the same six neurons. The results are shown in Figure 5. Note that the slopes in the Figure correspond to *H*. It is observed that the lines are quite straight, therefore, the power-law relation of equation (11) is well defined. More interestingly, it is observed that three neurons, shown in Figures 5A–C, are characterized by a single fractal scaling, with the Hurst parameter ranging from about 0.5 to about 0.73, indicating no correlations or weak correlations. On the other hand, the three other neurons shown in Figures 5D–F have very large Hurst parameters, indicating very strong temporal LRD. Note that the AFA curves for those three neurons present a turning point (called fractal scaling break) at around *m* = 2^6^ ∼ 2^7^, which corresponds to about 6.4 ∼ 12.8 s. Interestingly, the time scale of 6.4 ∼ 12.8 s is comparable to the average time of 8 s between two successive reaching tasks. These two features, a large Hurst parameter, and fractal scaling break at the time scale comparable to the average time between two successive reaching tasks, suggest that neurons well correlated with hand trajectory experienced a “re-setting” effect at the start of each reaching task, in the sense that within the movement correlated neurons the spike trains’ LRD persisted about the length of time the monkey used to switch between task executions. A new task execution re-sets their activity, making them only weakly correlated with their prior activities on longer time scales. This necessitates that a difference in the detail of long-range dependence must come into being with the new task execution, breaking the LRD associated with the prior task.

### 3.6 LRD in neuronal firings revealed by wavelet analysis

**Figure 6:**
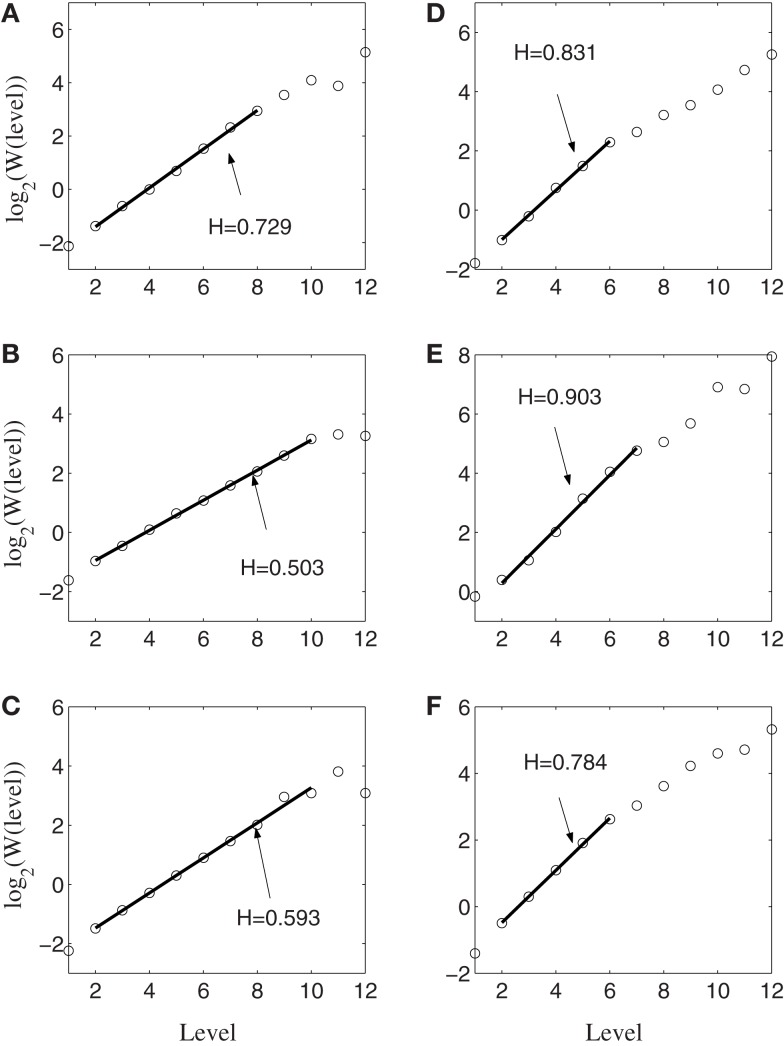
**Wavelet analysis of the same six neurons**. Here, the slope equals *H*. Notice the consistency with AFA analysis.

Figure 6 shows the result of wavelet analysis of the spike counting data of the same six neurons. We observe that the results are consistent with those based on AFA. Therefore, we can conclude that firings of the neurons well correlated with hand movements are characterized by large *H* with a scaling range up to the average time between two successive reaching tasks.

### 3.7 Multifractal AFA and wavelet analysis of superimposed neuronal firings

**Figure 7:**
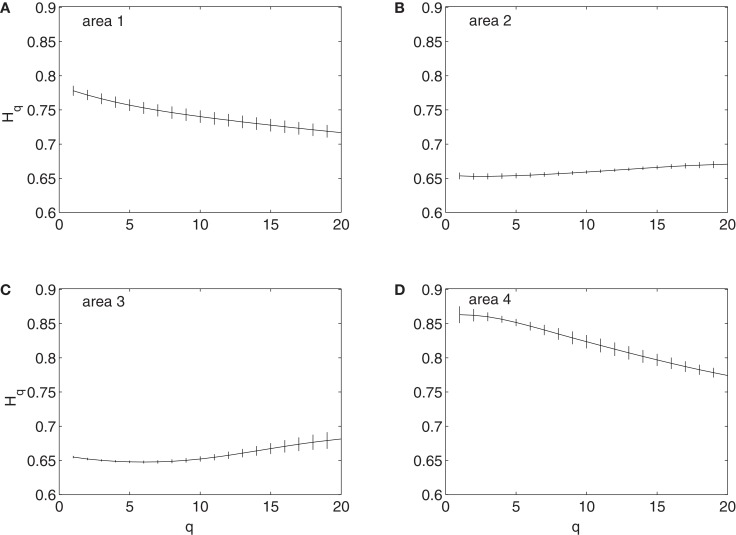
**MF-AFA of the “superimposed” spike train in the four brain areas, where (A) for area 1, left posterior parietal (PP); (B) area 2, left primary motor (MI); (C) area 3, left dorsal premotor (PMD); and (D) area 4, right primary motor and dorsal premotor (MI/PMD)**.

**Figure 8:**
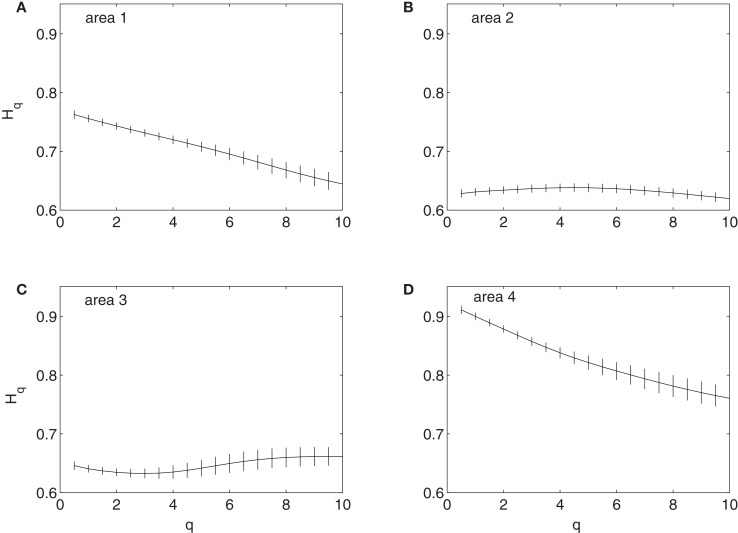
**MF wavelet analysis of the “superimposed” spike train in the four brain areas, where (A) for area 1, left posterior parietal (PP); (B) area 2, left primary motor (MI); (C) area 3, left dorsal premotor (PMD); and (D) area 4, right primary motor and dorsal premotor (MI/PMD)**.

To examine whether the neurons in the four brain areas (Table 1) may have different fractal properties, we combined the neuronal firings in each brain area and obtained a “superimposed” spike train, and then performed multifractal analysis based on MF-AFA and multifractal wavelet analysis. The results are shown in Figures 7 and 8, respectively. Recalling that multifractal is characterized by a non-constant *H*(*q*), while a monofractal is characterized by a fairly constant *H*(*q*), we conclude that areas 1 and 4 show multifractal behavior, while areas 2 and 3 show weak multifractal or monofractal behavior. This suggests that different brain areas may have different roles in coordinating movements.

### 3.8 Important vs. irrelevant neurons

Although 104 neurons have been analyzed here, there are only slightly more than 10 neurons that exhibit the interesting fractal scalings discussed above, and thus can be classified as important neurons. Amazingly, when those neurons are used to train a Wiener filter, the simulated trajectory already achieves about 65% correlation with the actual hand trajectory. However, when similar number of other neurons are used to predict a trajectory, the correlation between the predicted trajectory and the actual trajectory becomes very poor. Therefore, those neurons are indeed the most important in reaching tasks.

## 4 Discussions

To better understand the causal relation between neural inputs and movements, in this study, we have employed three different types of fractal and multifractal techniques, including Fano factor analysis, multifractal adaptive fluctuation analysis (MF-AFA), and wavelet multifractal analysis, to study whether neuronal firings related to movements may have LRD. We find that Fano factor analysis always indicates LRD in neuronal firings, irrespective of whether those firings are correlated with movement trajectory or not. Therefore, Fano factor analysis, while indicating that neuronal firings related to movements are generally non-Poisson, does not reveal any actual correlations between neural inputs and movements. This may be due to the overwhelming non-stationarity nature of neuronal firings. More interestingly, we have found that MF-AFA and wavelet multifractal analysis can clearly indicate that when neuronal firings are not well correlated with movement trajectory, they only have weak or very short-range temporal correlations. When neuronal firings are well correlated with movements, they are characterized by very strong temporal correlations, up to a time scale comparable to the average time between two successive reaching tasks. Beyond that time scale, LRD no longer exists. This suggests that neurons well correlated with hand trajectory experienced a re-setting effect at the start of each reaching task, in the sense that within the movement correlated neurons the spike trains’ LRD persisted about the length of time the monkey used to switch between task executions. A new task execution re-sets their activity, making them only weakly correlated with their prior activities on longer time scales. This necessitates that a difference in the detail of long-range dependence must come into being with the new task execution, breaking the LRD associated with the prior task.

The existence of a group of neurons whose firings are well correlated with hand movement suggests that neural information processing is carried out by a *dynamic coalition of neurons*, as hypothesized by Edelman and Tononi (59), Crick and Koch (60), Gao et al. (57), and Furstenau (61). By a *coalition of neurons*, it is meant that the coalition involves many types of excitatory and inhibitory interconnected neurons, which change the activities of their fellow members. By *dynamic*, it is meant that neurons within the coalition may leave the coalition and not participate in neural information processing, such as controlling hand movements. After they leave the coalition, they may re-join the coalition later; or new neurons can join the coalition. To better understand these statements, we note that in the time interval of three hand movements shown in Figure 1, *if we define the dynamic coalition of neurons by their correlation with the hand trajectory, then neuron 1 does not belong to the coalition, neuron 5 belongs to the coalition, and neurons 2 to 4 are transient members – they do not belong to the coalition at the 1st, 2nd, and 3rd hand movement, respectively.* This dynamic aspect is also responsible for the re-setting aspect of the important neurons discussed earlier. While the process of leaving and joining the coalition makes the structure of the network of the coalition highly time-varying, the collective behavior of the coalition must be fairly stable, since it controls movements. It is interesting to note that the notion of soft assemble proposed by Turvey (62) and the hypothesis of self-organization perception and action proposed by Van Orden et al. (63) are consistent with the framework discussed here.

The above discussion highly suggests that understanding the collective behavior of the coalition of neurons will be critical for fully realizing the promises of BMIs. One testable idea is to select neurons having relatively large Hurst parameters for adaptation algorithms thereby reducing the number of free parameters. Our own experience, as described in the last section, suggests that this is a valid idea. This is also consistent with the work of Sanchez et al. (26). The best predictions by adaptive algorithms may be obtained by not only using the notion of LRD, but also the notion of “dynamic” or “non-stationary.” By the latter, we mean that when a neuron has left the coalition of neurons performing a desired task, it then should be removed in the adaptive algorithm for prediction.

## Conflict of Interest Statement

The authors declare that the research was conducted in the absence of any commercial or financial relationships that could be construed as a potential conflict of interest.
